# Draft Genome Sequence of *Fusarium* sp. Strain DS 682, a Novel Fungal Isolate from the Grass Rhizosphere

**DOI:** 10.1128/MRA.00884-20

**Published:** 2021-01-07

**Authors:** Arunima Bhattacharjee, Lindsey N. Anderson, Trinidad Alfaro, Andrea Porras-Alfaro, Ari Jumpponen, Kirsten S. Hofmockel, Janet K. Jansson, Christopher R. Anderton, William C. Nelson

**Affiliations:** a Earth and Biological Sciences Directorate, Pacific Northwest National Laboratory, Richland, Washington, USA; b Department of Ecology, Evolution, and Organismal Biology, Iowa State University, Ames, Iowa, USA; c Department of Biological Sciences, Western Illinois University, Macomb, Illinois, USA; d Division of Biology, Kansas State University, Manhattan, Kansas, USA; University of California—Riverside

## Abstract

The novel fungal strain, *Fusarium* sp. strain DS 682, was isolated from the rhizosphere of the perennial grass, *Bouteloua gracilis*, at the Konza Prairie Biological Station in Kansas. This fungal strain is common across North American grasslands and is resilient to environmental fluctuations. The draft genome is estimated to be 97.2% complete.

## ANNOUNCEMENT

*Fusarium* sp. strain DS 682 (DS 682) was isolated from the rhizosphere of *Bouteloua gracilis*, a blue grama perennial warm season (C4) grass, at the Konza Prairie Biological Station (KPBS) in Kansas. Specifically, the strain was isolated from field site 4A as part of a study to evaluate the importance of climatic, edaphic, and host plant traits as predictors of rhizobiome community structure (1). The isolation process included surface sterilization of excised root tissue of *B. gracilis* and plating onto malt extract agar (MEA; Difco Laboratories, MD) amended with 50 mg/liter streptomycin and tetracycline. After a 14-day incubation at room temperature, emerging colonies with different morphologies were transferred onto fresh MEA (supplemented with antibiotics) to establish pure cultures.

DS 682 was chosen for whole-genome sequencing because it is common to soil and grass rhizospheres across North America, demonstrating metabolic adaptability to environmental fluctuations. The fungal isolate was identified as a *Fusarium* sp. by amplicon sequencing of the internal transcribed spacer (ITS) region using the primers ITS1F (2) and ITS4 (3) and the Web resource FUSARIUM-ID (http://isolate.fusariumdb.org/blast.php). This identification was verified and refined using additional genomic markers (see below).

Genomic DNA was prepared from DS 682 mycelium grown on MEA. Cells were lysed via sonication (Branson SFX 150; Emerson Electric, MO) and extracted by using a DNeasy PowerSoil kit (Qiagen, Inc., Germany). DNA libraries were prepared by using a NEBNext Ultra DNA library prep kit (Illumina, San Diego, CA) and validated by using a DNA 1000 Chip on the Agilent TapeStation (Agilent Technologies, CA). Sequencing was performed on the Illumina HiSeq X platform (150 bp, paired end) by Genewiz, Inc. (South Plainfield, NJ).

Sequence reads were assembled using the ATLAS pipeline (4). Briefly, reads were deduplicated using Clumpify (BBTools v37.78), quality filtered using BBDukF, and decontaminated by searching against PhiX and rRNA reference data sets using BBSplit. Quality-filtered reads were normalized using KmerNormalize, error corrected using Tadpole, and merged using BBMerge. Assembly was performed by MEGAHIT (v1.1.2) (5), resulting in 13,284 contigs, totaling 56.5 Mbp and ranging in length from 200 to 215,770 bp (average, 4,253 bp; *N*_50_, 24,701 bp). The average coverage was determined to be 233-fold by QUAST (v5.0.2) (6). Genemark-ES (v4.59) was used to predict coding genes, resulting in 19,949 predicted proteins, of which 8,849 were assigned to a KEGG Orthology (KO) family using kofamscan (v1.3). Genome completeness was estimated to be 97.2% with low duplication and low fragment count (C:97.2%[S:95.7%,D:1.5%],F:1.7%,M:1.1%,n:4494) by BUSCO (v4.0.6) using the hypocreales_odb10 reference gene set.

**FIG 1:**
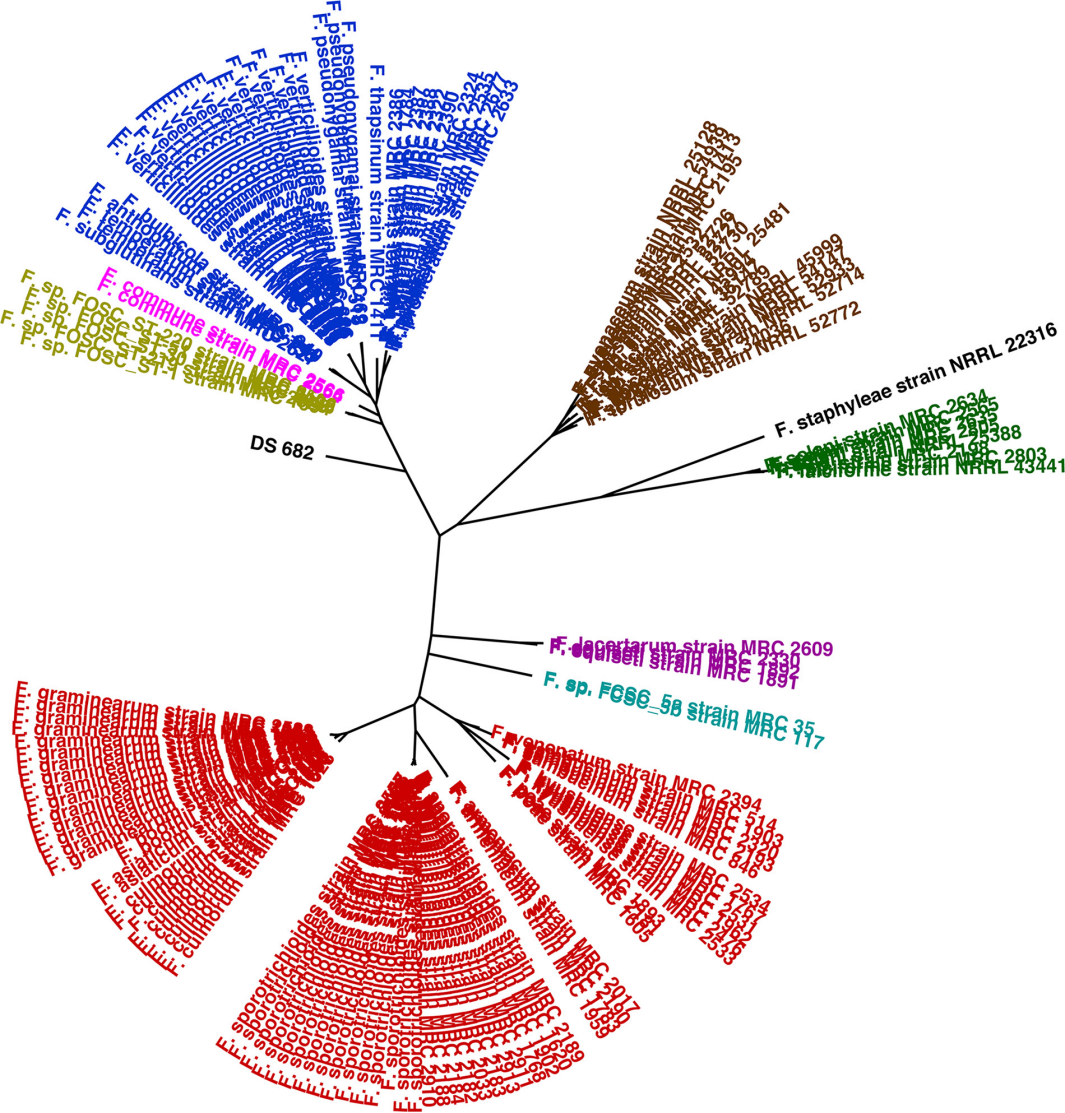
Molecular phylogeny of *Fusarium* sp. strain DS 682. Sequences of the RNA polymerase II (RPB2) and translation elongation factor 1 (TEF1) genes were concatenated and aligned using MUSCLE v3.8.425 (9). An approximate maximum-likelihood tree was generated using FastTree (10) and visualized using FigTree v1.43 (http://tree.bio.ed.ac.uk/software/figtree/). Label colors denote species complex memberships: red, *F. sambucinum* species complex (FSAMSC); purple, *F. incarnatum-equiseti* species complex (FIESC); cyan, *F. chlamydosporum* species complex (FCSC); mustard, F. oxysporum species complex (FOSC); blue, *F. fujikuroi* species complex (FFSC); brown, *F. tricinctum* species complex (FTSC); green, F. solani species complex (FSSC); magenta, *F. nisikadoi* species complex (FNSC); black, none assigned.

Phylogenetic placement was determined by analyzing the genes for RNA polymerase II (RPB2) and translation elongation factor I (TEF1). The data set published by O’Donnell et al. (7) was used as a reference database. Sequences were imported into the AliView (v1.19) (8) interface, aligned using MUSCLE v3.8.425 (9), and clustered using FastTree v2.2.11 (10) with the “nt” and “gtr” options. This analysis suggests that DS 682 represents a novel lineage within the *Fusaria* basal to the *F. fujikuroi*, *F. nisikadoi*, and F. oxysporum species complexes (Fig. 1).

Comparison with Fusarium oxysporum revealed 4,934 genes (∼25% of the genome) in DS 682 that did not have close similarity (amino acid similarity > 60%, E value ≤ 10^–10^). Of these, only 664 were assigned to 355 KO families with diverse activities.

### Data availability.

Sequence data have been deposited at the PNNL DataHUB repository (https://data.pnnl.gov/about) and are available for download under project data DOI accession number 10.25584/KS4AIsoGFspDS682/1635527. The version described here is the first version. The data package contains raw reads, assembly, functional annotations, MIGS.eu.soil.5.0 metadata information, and a package “Read Me” file. The genome sequence information has also been deposited under BioProject number PRJNA664411 and under BioSample accession number SAMN16213216, and this whole-genome shotgun project has been deposited at DDBJ/ENA/GenBank under accession number JACYFE000000000, where the version described here is version JACYFE010000000. Gene models are available at https://www.ncbi.nlm.nih.gov/protein?LinkName=nuccore_protein_wgs&from_uid=1910285265.

## References

[1] Jumpponen A, Herrera J, Porras-Alfaro A, Rudgers J. 2017. Biogeography of root-associated fungal endophytes, p 195–222. *In* Tedersoo L (ed), Biogeography of mycorrhizal symbiosis. Springer International Publishing, Cham, Switzerland.

[2] Gardes M, Bruns TD. 1993. ITS primers with enhanced specificity for basidiomycetes - application to the identification of mycorrhizae and rusts. Mol Ecol 2:113–118. doi:10.1111/j.1365-294x.1993.tb00005.x. 8180733

[3] White TJ, Bruns T, Lee S, Taylor J. 1990. Amplification and direct sequencing of fungal ribosomal RNA genes for phylogenetics, p 315–322. *In* Innis MA, Gelfand DH, Sninsky JJ, White TJ (ed), PCR protocols. Academic Press, San Diego, CA.

[4] Kieser S, Brown J, Zdobnov EM, Trajkovski M, McCue LA. 2020. ATLAS: a Snakemake workflow for assembly, annotation, and genomic binning of metagenome sequence data. BMC Bioinformatics 21:257. doi:10.1186/s12859-020-03585-4. 32571209 PMC7310028

[5] Li D, Luo R, Liu C-M, Leung C-M, Ting H-F, Sadakane K, Yamashita H, Lam T-W. 2016. MEGAHIT v1.0: a fast and scalable metagenome assembler driven by advanced methodologies and community practices. Methods 102:3–11. doi:10.1016/j.ymeth.2016.02.020. 27012178

[6] Gurevich A, Saveliev V, Vyahhi N, Tesler G. 2013. QUAST: quality assessment tool for genome assemblies. Bioinformatics 29:1072–1075. doi:10.1093/bioinformatics/btt086. 23422339 PMC3624806

[7] O’Donnell K, McCormick SP, Busman M, Proctor RH, Ward TJ, Doehring G, Geiser DM, Alberts JF, Rheeder JP. 2018. Marasas et al. 1984 “Toxigenic *Fusarium* Species: Identity and Mycotoxicology” revisited. Mycologia 110:1058–1080. doi:10.1080/00275514.2018.1519773. 30481135

[8] Larsson A. 2014. AliView: a fast and lightweight alignment viewer and editor for large datasets. Bioinformatics 30:3276–3278. doi:10.1093/bioinformatics/btu531. 25095880 PMC4221126

[9] Edgar RC. 2004. MUSCLE: a multiple sequence alignment method with reduced time and space complexity. BMC Bioinformatics 5:113. doi:10.1186/1471-2105-5-113. 15318951 PMC517706

[10] Price MN, Dehal PS, Arkin AP. 2010. FastTree 2: approximate maximum-likelihood trees for large alignments. PLoS One 5:e9490. doi:10.1371/journal.pone.0009490. 20224823 PMC2835736

